# Exploring the interplay between circadian rhythms and prostate cancer: insights into androgen receptor signaling and therapeutic opportunities

**DOI:** 10.3389/fcell.2024.1421204

**Published:** 2024-07-01

**Authors:** Hongyan Xia, Yang Zhan, Li Wang, Xiaohui Wang

**Affiliations:** ^1^ College of Basic Medical Sciences, Shanxi Medical University, Taiyuan, China; ^2^ National Engineering Laboratory for AIDS Vaccine, College of Life Sciences, Jilin University, Changchun, China; ^3^ Department of Pathology, Shanxi Medical University, Taiyuan, China

**Keywords:** circadian rhythm, chronotherapy, circadian clock, circadian genes, prostate cancer, AR signaling pathway

## Abstract

Circadian rhythm disruption is closely related to increased incidence of prostate cancer. Incorporating circadian rhythms into the study of prostate cancer pathogenesis can provide a more comprehensive understanding of the causes of cancer and offer new options for precise treatment. Therefore, this article comprehensively summarizes the epidemiology of prostate cancer, expounds the contradictory relationship between circadian rhythm disorders and prostate cancer risk, and elucidates the relationship between circadian rhythm regulators and the incidence of prostate cancer. Importantly, this article also focuses on the correlation between circadian rhythms and androgen receptor signaling pathways, as well as the applicability of time therapy in prostate cancer. This may prove significant in enhancing the clinical treatment of prostate cancer.

## 1 Introduction

Prostate cancer is the second most common cancer worldwide, and it is expected that two million men will suffer from prostate cancer by 2040 (Sandhu et al., 2021; Siegel et al., 2022). The pathogenesis of prostate cancer is currently unclear, but it is closely related to advancing age and hormone secretion. The standard treatment for clinical prostate cancer is androgen deprivation therapy (ADT), targeting androgens and androgen receptors, with good initial efficacy and a 5-year survival rate of 100%. However, drug resistance gradually emerges, and the survival rate of recurrent or metastatic prostate cancer hovers around 30% (Ku et al., 2019; Jamroze et al., 2021). Currently, no better treatment options are available. Therefore, simply targeting androgens and androgen receptor signaling pathways cannot block tumor development, and it is necessary to explore other therapeutic directions to enhance clinical treatment efficacy.

Research has shown that the occurrence of prostate cancer is regulated by multiple factors, including immutable factors (such as race and genetics) and variable factors (such as hormones, age, and occupational environment) (Gandaglia et al., 2021; Bergengren et al., 2023). Therefore, identifying modifiable factors that can be used as cancer prevention targets remains a considerable challenge (Barul et al., 2019). Several decades ago, researchers used light-at-night simulations to explain, to a certain extent, the high risk of breast and prostate cancer in industrialized societies (Stevens, 1987; Stevens et al., 1992). However, in 2007, the International Agency for Research on Cancer (IARC) classified “work involving day and night shifts” as a human carcinogenic factor based on adequate animal experiments and limited population sample analysis (Straif et al., 2007). Increasing research confirms that circadian rhythm disorders are closely linked to the development of tumors. Androgens are necessary for early prostate cancer growth, and their secretion is regulated by the circadian rhythm, with the highest levels occurring in the morning and the lowest in the evening; this rhythmicity disappears with age (Cooke et al., 1993). This indicates that circadian rhythm regulation may be an effective way to enhance the therapeutic effect of prostate cancer.

This article summarizes research in recent years that has emphasized the importance of the relationship between the biological clock and prostate cancer. We searched for keywords such as “circadian cycle”, “prostate tumor”, “cancer”, “androgen”, “night work”, “rotation”, “circadian rhythm genes”, “clock genes”, “circadian rhythm”, and “clock gene polymorphism” in MEDLINE, Scopus, and PubMed databases, and summarized articles highlighting the relationship between prostate and circadian rhythm. We hope to provide a theoretical basis for the treatment of prostate cancer from an alternative perspective.

## 2 Circadian rhythms

The circadian rhythm is a natural biological clock system composed of neurons and genetic networks that control 24-h cyclical changes in the body to adapt to changes in the environment (Dollish et al., 2024). Among external environmental factors, light is the main signal of the circadian rhythm, and the Suprachiasmatic Nucleus (SCN) in the brain receives light signals from the optic nerve and adjusts the biological clock to synchronize with light by releasing neurotransmitters and conductive cell signals. The circadian rhythm regulates a variety of physiological functions including sleep-wake cycles, body temperature, hormone secretion, cognitive function, and the immune system. It also maximizes the adaptability of organisms to their external environment in order to maintain internal stability (Villanueva-Carmona et al., 2023; Zhang et al., 2023; Kanan et al., 2024).

**FIGURE 1:**
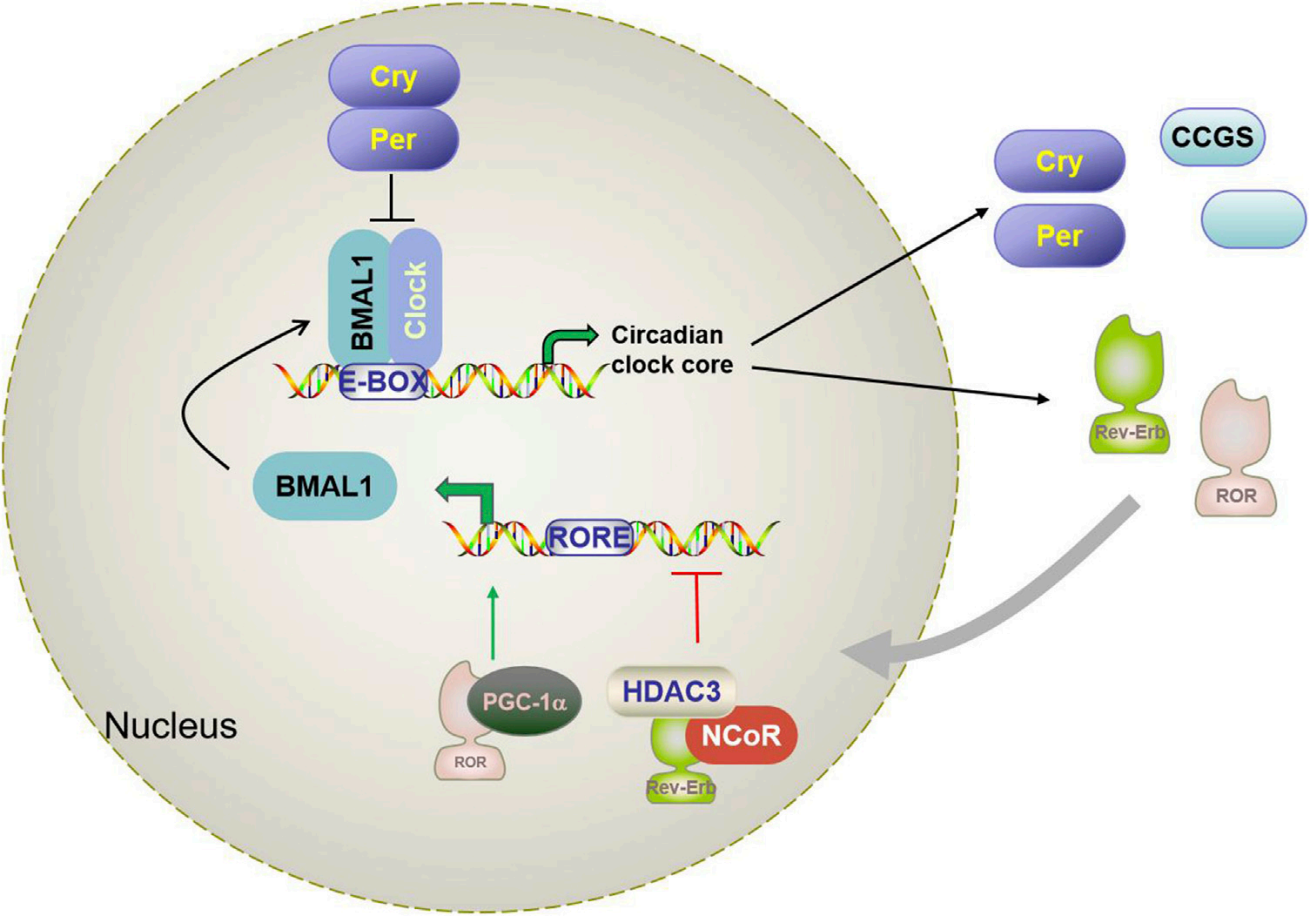
Schematic diagram of the mammalian circadian clock transcriptional feedback loop. Bmal1 and CLOCK form a heterodimer that binds to the E-box in the downstream gene promoter region and activates Per, CRY, RORα, Rev-erbα, and CCGs. CRY and Per constitute the inhibition arm of TTPL; they dimerize and enter the nucleus to inhibit the activation of Bmal1-CLOCK. This results in oscillating patterns of gene expression. A secondary loop composed of REV-ERBα and RORα competitively binds to the RRE binding site in the BMAL1 promoter and regulates the transcription level of Bmal1. Brain and muscle aryl hydrocarbon receptor nuclear transporter-like protein 1 (Bmal1); Circadian locomotor output cycles kaput (CLOCK); Cryptochrome (Cry); Period (Per); reverse strand of ERB protein alpha (Rev-erbα); Orphan retinoic acid receptor-related α; CCG(clock-controlled gene).

The circadian rhythm is a complex biological process involving multidimensional biological mechanisms. In mammalian cells, the circadian system is composed of core clock genes and proteins that are regulated by a transcription-translation-feedback loop (TTFL) network (Reppert and Weaver, 2002) (shown in Figure 1). Basic helix-loop-helix ARNT-like protein 1 or aryl hydrocarbon receptor nuclear translocator-like protein 1 (Bmal1/ARNTL), and circadian locomotor output cycles kaput (CLOCK) form heterodimers that bind to the target gene promoter E-BOX (CACGTG). This initiates the transcription of target genes of specific circadian regulators (*per1* and *per2*) and cryptochromes (*CRY1* and *CRY2*) (Tsuchiya et al., 2020). Per and Cry proteins accumulate and form heterodimers, which are recruited through casein kinase 1d (CKd) to isolate BMAL1: CLOCK from E-BOX and inhibit their transcriptional activity (Battaglin et al., 2021a; Cao et al., 2021). The second feedback circuit of the regulatory network involves protein adjustment of the orphan receptors RORα/β/γ and REV-ERBα/β (as shown in Figure 1). RORs and REV-ERBs competitively bind to ROR response elements (RORE) in the *Bmal1* promoter region. RORs activate *Bmal1* transcription, while REV-ERBs inhibit *Bmal1* transcription with nuclear receptor co-suppressor NcoR. BMAL1:CLOCK also controls *ROR* and *REV-ERB* transcripts through binding the E-BOX of *ROR*s and *REV-ERB*s promoter regions (Hernandez-Rosas et al., 2020; Battaglin et al., 2021; Ruan et al., 2021). In addition to the above core clock genes, there are also a number of clock-controlled gene (CCGs) that also contain e-boxes, which are also directly regulated by the BMAL1:CLOCK dimer and are involved in the daily rhythm of clock output and various physiological processes.

However, due to temporal variations, rotation work, abnormal dietary patterns, and lifestyle changes, circadian rhythms may be disrupted or disturbed, causing circadian rhythm disruption (CRD). This is closely associated with a variety of diseases including cardiovascular disease, autoimmune disease, obesity, and cancer (Fishbein et al., 2021). In general, the clock rhythm is a timing system that helps organisms adapt to changes in the environment and maintain normal rhythms and functions. Its mechanisms of action involve gene regulation, optical signal transmission, hormone secretion and a variety of physiological effects.

## 3 Epidemiological evidence of rhythm disorders and prostate cancer

In order to further clarify the association between circadian rhythm disturbance and prostate cancer risk, a number of epidemiological studies have explored the association between night work/sleep disorders and prostate cancer. However, the results are somewhat contradictory (as shown in Table 1). Some studies have shown that circadian rhythm disturbance caused by shift work is associated with an increased risk of prostate cancer. Full-time shifts increased the risk of prostate cancer by 34 percent (Conlon et al., 2007). Recall studies of pilots in Nordic countries show that male pilots older than 60 years have a correlated risk of prostate cancer with flight time (Pukkala et al., 2003). A retrospective cohort study and prospective studies from Japan showed that age adjustment significantly increases the risk of illness for shift workers when compared to daily workers (RR adjusted for age = 3.0, 95% CI = 1.2, 7.3); an effect un-impacted by factors such as weight, alcohol consumption, or smoking (Kubo et al., 2006; Kubo et al., 2011). Moreover, a population case study in Canada, conducted between 1979 and 1985 that analyzed 3,137 male cancer patients and 512 counterparts, revealed that nighttime staff members had a threefold increased prostate cancer risk (RR = 2.77, 95% CI = 1.96, 3.92) (Parent et al., 2012). These studies all suggest that circadian rhythm disturbance increases the risk of prostate cancer. On the contrary, some studies suggest that circadian rhythm disturbance may not be associated with increased cancer risk. A Canadian population case survey from 2005 to 2012, which included 1904 prostate cancer patients and 1965 healthy controls, showed that there was no correlation between the risk of disease and working hours or working years. A workforce analysis of males (N = 1319, SIR 1.04, 95% CI 0.99–1.10) and females (N = 70 patients, SIR 0.94, 95% CI 0.74–1.18) revealed no associations between shift work and the risk of developing breast or prostate cancer (Schwartzbaum et al., 2007). Similarly, population analysis in the U.S. also showed that rhythm disorders caused by work patterns and sleep time are not associated with an increased risk of prostate cancer (Gapstur et al., 2014). However, population analysis in Spain once again revealed that workers who work night shifts for more than a year have a significantly increased risk of prostate cancer, and the risk ratio is positively correlated with exposure time (Papantoniou et al., 2015a). A recent meta-analysis of three case-control and five cohort studies showed a significantly increased risk of prostate cancer in night-time workers (RR: 1.24, 95% CI: 1.05-1.46; *p* = 0.011) (DeBono et al., 2023). Although the available results are inconsistent, due to differences in data sources and data processing methods, it can still be noted that there is a certain correlation between altered circadian rhythm and prostate cancer risk.

**TABLE 1:** Epidemiologic evidence of rhythm disturbance and prostate cancer.

Countries and regions	OR/SIR	95% CI	Age	No. of control	No. of cases	Results
Northeastern Ontario Conlon et al. (2007)	1.34	1.0, 1.8	45–84	1632	760	Starting full-time shift work 37–44 years before diagnosis is associated with a 34% increase in risk
Northern Europe Pukkala et al. (2003)	1.56	0.67,3.07	>60	456	366	The risk of prostate cancer is positively correlated with flight time
Japan Kubo et al. (2011)	1.79	0.57,5.68	mean age 55.5	4168	827	Workers involved in shift work had a significantly increased risk of illness
Japan Kubo et al. (2006)	3.0	1.2,7.3	45–79	89179	14523	Shift workers had a significantly increased age-adjusted relative risk compared to day workers
Canada Parent et al. (2012)	2.77	1.96,3.92	mean age:59–63	512	3137	Night workers have a three-fold increased risk of prostate cancer
Swedish Schwartzbaum et al. (2007)	1.04	0.99,1.00	All ages	2102126	1319	Shift work did not increase the risk of cancer
Spain Papantoniou et al. (2015a)	1.14	0.94,1.37	27–85	1388	1095	a significant association between night shift work and prostate cancer, especially with tumors with a poorer prognosis
America Gapstur et al. (2014)	1.08	0.95,1.22	Mean age 52	274,702	18,126	Work schedule and insomnia frequency were not associated with risk of fatal prostate cancer

OR, odds ratio.

SIR, standardized incidence ratio.

95% CI, 95% confidence interval.

## 4 Circadian rhythm gene and prostate cancer risk

Epidemiological studies have confirmed a certain correlation between circadian rhythm disturbance and the risk of prostate cancer, providing a new option for cancer prevention, diagnosis and treatment (Sulli et al., 2019). Researchers have explored the relationship between clock gene polymorphisms and cancer, and identified an association between rhythm genes and tumor occurrence and development (Grundy et al., 2013; Zienolddiny et al., 2013; Benna et al., 2017; Mocellin et al., 2018). However, there are only seven relevant studies on prostate cancer at present, and although few, they can still inform the correlation between rhythm genes and prostate cancer (as show in Table 2).

**TABLE 2:** Gene single nucleotide polymorphisms (SNP) And Prostate Cancer Risk.

Gene	SNP-ID	Relationship with PCa risk	References
*CRY2* *CSNK1E* *NPAS2* *PER1* *PER3*	rs1401417 rs1005473 rs2305160 rs2585405 54-bp repeated length variants	NPAS2 variant A genes with a lower risk of prostate cancer; CRY2 SNP increases the risk of disease by 1.7 times	Chu et al. (2008a)
*NPAS2*	rs746924	Suggestive association with the risk for prostate cancer	Gu et al. (2017), Chu et al. (2018), Mocellin et al. (2018)
*ARNTL, RORA, RORB, NR1D1, PER3, and CLOCK*	_	Are associated with prostate cancer	Mocellin et al. (2018)
*PER1* *PER2* *PER3* *CSNK1E* *CRY1* *CRY2* *ARNTL* *CLOCK* *NPAS2*	rs885747 and rs2289591; rs7602358; rs1012477; rs1534891; rs12315175; rs2292912; rs7950226; rs11133373; rs1369481, rs895521, and rs17024926	Significantly correlated with prostate cancer susceptibility	Zhu et al. (2009)
*PER1* *PER3* *CLOCK*	rs885747 and rs2289591; rs1012477; rs11133373	Were more prone to tumor invasion	Zhu et al. (2009)
*CRY1*	rs7297614 and rs1921126	Is correlated with prostate cancer risk	Markt et al. (2015)
*PER1 and NPAS2*	_	are related to all prostate cancers	Wendeu-Foyet et al. (2019)
*RORA*	_	is significant for invasive tumors	Wendeu-Foyet et al. (2019)

As the largest circadian rhythm gene, *NPAS2* is paralogous to the CLOCK protein, and can replace the function of CLOCK, forming heterodimers with BMAL1 to regulate circadian rhythm (Landgraf et al., 2016). In order to further explore the relationship between *NPAS2* and prostate cancer, a study by Chu *et al.* compared the association between 240 circadian rhythm gene single nucleotide polymorphisms (SNPs) and prostate cancer risk in 450 patients treated with finasteride (an androgen biological activation inhibitor) and 422 controls. In the finasteride group, the *NPAS2* variant (rs746924) was associated with an increased associated risk of prostate cancer (Chu et al., 2018). Two other studies were evaluated at gene level and pathway analysis confirmed the correlation between *NPAS2* and prostate cancer (Gu et al., 2017; Mocellin et al., 2018). In addition, experimental research also confirmed that *NPAS2* expression is increased in tissue from prostate cancer patients when compared to healthy prostate tissue. NPAS2 increases the expression of hypoxia inducible factor-1A (HIF-1a), leading to enhanced glycolytic metabolism in prostate cancer thereby promoting tumor growth (Ma et al., 2023). This provides a new research direction for exploring the mechanism of metabolic reprogramming in prostate cancer cells.

A 2008 population-based study analyzed five SNPs of five Circadian rhythm genes, namely, *CRY2* rs1401417:G>C, *CSNK1E* rs1005473:C>A, *NPAS2* rs2305160:G < A, *PER1* rs2585405:G>C and *PER3* 54-bp repeat length variants. *NPAS2* variant A genes were associated with a lower risk of prostate cancer (ratio = 0.5, 95% CI, 0.3.0-1.0) in men with mild insulin resistance when compared to men with the GG genotype. Additionally, these genes significantly increased the risk of male prostate cancer with *CRY2*-C variation-level genes by 1.7 times (95% CI, 1.1-2.7) (Chu et al., 2008a). Another SNP study in men from the United States of the Caucasus (including 1,308 cases and 1,266 counterparts) included nine rhythmic genes. For example, rs885747 and rs2289591 in PER1; rs7602358 in PER2; rs1012477 in PER3; rs1534891 in CSNK1E; rs12315175 in CRY1; rs2292912 in CRY2; rs7950226 in ARNTL; rs11133373 in CLOCK; and rs1369481, rs895521, and rs17024926 in NPAS2 were significantly correlated with prostate cancer susceptibility, while four SNPs, rs885747 and rs2289591 in PER1; and rs1012477 in PER3 and rs11133373 in CLOCK, were more prone to tumor invasion (Zhu et al., 2009). Although the above statistical analyses yielded different results, they all highlight the correlation between *PER3* and prostate cancer, making it particularly important to explore its mechanism of action. Research has shown that *PER3* is downregulated in prostate cancer stem cells (PCSS), which in turn enhances their tumorigenicity and ability to form spheroids and colonies in the host body. PER3 is also associated with paclitaxel resistance in prostate cancer (Li et al., 2021). Clinical case analysis showed that PER3 expression in the paclitaxel resistant group is lower than that in the non-resistant group. Moreover, overexpression of PER3 in resistant cells can lead to a decrease in Notch1 expression, cell cycle arrest, and weakened resistance of paclitaxel resistant cells (Cai et al., 2018).

In addition, a study assessed the association of 86 SNPs of circadian rhythm genes with the risk of fatal prostate cancer. Findings showed that all core circadian rhythm gene pathways, except *CRY1* (rs7297614 and rs1921126), were not correlated with prostate cancer risk (Markt et al., 2015). However, in the path analysis report by Mocellin *et al.*, genes such as *ARNTL, RORA, RORB, NR1D1, PER3,* and *CLOCK* were associated with prostate cancer (Mocellin et al., 2018). Considering the limited number of samples and the number of SNPs, the 2019 study included 1515 participants (approximately 89%) from the EPICAP study population, expanding the list of clock rhythm genes (31 of whom) and including large numbers of SNPs (872). This study achieved high coverage of rhythmic genetic variations, maximized the error classification of genotypes, and combined analysis of genetic polymorphisms and signaling pathways (Wendeu-Foyet et al., 2019). Findings confirmed that core clock rhythm pathways were associated with prostate cancer. *PER1* and *NPAS2* levels were related to all prostate cancers, while only *RORA* was significant for invasive tumors (Wendeu-Foyet et al., 2019). The above studies provide evidence for the potential link between genetic variation in clock rhythm genes and the risk of prostate cancer, but further exploration of the mechanisms involved in the relationship between these genes and prostate cancer is still needed.

## 5 Circadian rhythm and androgen receptor (AR) signaling pathways

### 5.1 The role of AR signaling pathways in prostate cancer

**FIGURE 2:**
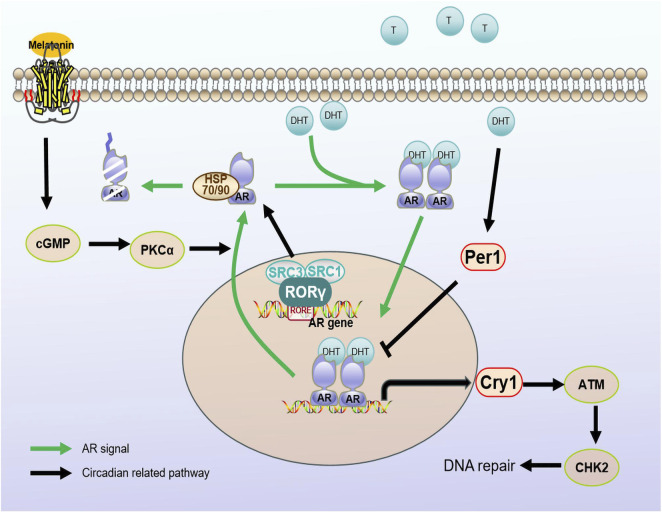
Regulatory mechanisms between circadian regulators and AR signaling pathways. Androgen receptors are stimulated by androgens into the nucleus. Melatonin, as a hormonal regulator of clock rhythm, promotes the nucleation process of AR through the cGMP/PKCα signaling pathway. The rhythm gene PER1 is positively regulated by androgens. When stimulated by DHT, PER1 may interact with the AR signaling pathway by influencing the expression of AR regulatory genes. At the same time, CRY1, as a target gene of the AR signaling pathway, regulates the temporal expression of DNA repair factor ATM/CHK2, promoting DNA damage repair and tumor growth. RORγ can recruit cofactors SRC3/SRC1 to directly regulate AR protein transcription. Androgen receptor (AR); dihydrotestosterone (DHT); Cryptochrome (Cry); Period (Per); orphan retinoic acid receptor-related alpha (RORα).

The androgen receptor (AR) is a member of the steroid family of nuclear transcription factors which comprises adhesive-dependent transcription factors. The open reading box of the *AR* gene contains eight exons that constitute four structural domains: the N-terminal domain encoding trans-activation function, the DNA binding domain (DBD), the hinge domain, and the ligand-binding domain (LBD) (Tan et al., 2015). Under normal conditions, the AR is present in the cytoplasm in an inactive state. It combines with heat shock proteins to form a complex. DHT stimulation causes the complex to dissociate, and the AR enters the nucleus, interacts with the androgen response element (ARE) in the promoter region of the target gene, and activates downstream gene transcription (Lallous et al., 2013; Tan et al., 2015) (as shown in Figure 2).

The role of the androgen receptor signaling pathway in prostate cancer has been widely proven, and the main mechanisms leading to tumor progression include mutations in the *AR* gene, synthesis of intra-tumoral androgens and abnormal AR proliferation and AR splicing. Approximately 50% of *AR* mutations are present in the LBD region, and studies have shown that *AR* mutation rates reach 20% in androgen-dependent tumors. For example, up to 50% of AR mutations in CRPC strains, such as residue 741 (W741C), can convert the AR antagonist bicalutamide to an activator that activates the AR and promotes tumor growth (Osguthorpe and Hagler, 2011). Furthermore, the F876L and H874Y/T877A mutations are resistant to enzalutamide and abiraterone, respectively (Yoshida et al., 2005; Azad et al., 2015). Although ADT therapy reduces testosterone levels in patients, non-testicular androgen synthesis occurs in CRPC patients. Non-steroidal generating enzymes such as 17βHSD3, AKR1C3 and SRD5A1 are involved in the non-classical synthesis of androgens, promoting tumoral androgen synthesis (Mohler et al., 2004; Chang et al., 2011). Androgen receptor splicing variants, such as ARV1, AR-V7/AR3 and AR-12/AR^v567es^, lead to truncated forms of the AR that lack ligand binding regions. Further, they do not require ligand activation and have sustained transcriptional activity (Sun et al., 2010; Chan et al., 2012). The most widely studied variant, AR-V7, has its own unique target gene and is also able to interact with the full-length AR (Antonarakis et al., 2014; Luo, 2016), promoting the activity of AR-FL and increasing patient resistance to hormone therapy.

### 5.2 Circadian rhythm regulator, androgen and the AR pathway

Several studies have shown that there are associations between prostate cancer incidence and androgen signaling and between prostate cancer incidence and circadian rhythm. The combination of rhythm regulation and AR signal targeting therapy may be an effective means of treating prostate cancer in future.

Androgens act mainly by activating androgen receptors to maintain secondary male characteristics, with 90% of androgens in the body being synthesized in the testicles and 10% by the adrenal cortex (Lindzey et al., 1994). Studies have shown that mutations in clock genes are significantly correlated with changes in serum sex hormone levels. In addition to their direct tumorigenic effect, androgens can promote the proliferation of prostate cancer cells (Chu et al., 2008b; Wittert, 2014). The secretion of androgens is regulated by the hypothalamus and has a rhythm, reaching a peak at 10 a.m. in the morning; an early morning peak that weakens with aging (Bremner et al., 1983; Cooke et al., 1993). Animal experiments have also shown that expression of the clock genes (Bmal1, Per, and Rev-erb) in older mice (18 months and 24 months) is also decreased compared to that in young mice (3 months) (Baburski et al., 2016). Other studies have confirmed that Bmal1, but not Per2 or Rev-erb, are downregulated by testosterone deficiency (Kawamura et al., 2014). This suggests that a decrease in clock genes may lead to circadian rhythm disorders in older males. According to our population analysis, peak androgenic hormone levels of night workers were also delayed compared to those of day workers (Papantoniou et al., 2015b). This may account for the potentially increased risk of prostate cancer in night workers. Approximately 1.5%–4.3% of genes in the human body are directly or indirectly related to androgen regulation. Rhythmic fluctuations in levels of AR-related mRNAs in prostate tissue (Cao et al., 2009; Wittert, 2014), suggest that ARs may associate the peripheral biological clock with hormone regulation.

Melatonin (N-acetyl-5-methoxytryptamine) is an endogenous indolamine that regulates the sleep-wake cycle and is an important clock rhythm hormone regulator. Its inhibitory effect depends on the GPCRs, MT1/Mel1A and MT2/Mel1B (Moretti et al., 2000; Xi et al., 2001). Multiple studies have demonstrated that melatonin can mediate the nuclear rejection of the AR in prostate cancer cells without inhibiting the binding capacity of androgens to the AR (Rimler et al., 2001; Rimler et al., 2002) (as shown in Figure 2). Follow-up studies further explored the mechanisms by which melatonin induces AR nuclear rejection, and the results showed that melatonin can increase cGMP levels in cells, activating PKC thereby leading to AR excretion. However, the addition of BAPTA, an intracellular calcium-bonding agent, can block the action of melatonin, confirming that the effect of melatonin depends on an increase in intracellular calcium (Lupowitz et al., 2001). Z Lupowitz *et al.* studied the members of the protein kinase C (PKC) subtype involved in AR nuclear rejection; for example, PKCα is expressed in the cytoplasm and undergoes membrane binding under the induction of melatonin, indicating that the activation of PKC is a key step in melatonin-mediated AR nuclear rejection (Sampson et al., 2006). Subsequent studies confirmed that melatonin inhibits the proliferation of androgen-sensitive LNCaP cells through the MT1 receptor both under androgen-free conditions and in naked mouse models (Xi et al., 2000; Xi et al., 2001). Moreover, melatonin enhances the effect of ADT by synergizing with MTI receptors, further blocking the growth of androgen-sensitive LNCaP tumor models. Interestingly, melatonin has limited inhibitory effects on androgen-independent prostate cancer cell lines PC-3 or DU145, possibly because the antiproliferative ability of MLT/MT1 depends on the activation of androgen/AR in prostate cancer cells (Siu et al., 2002). Studies by Liu and others have confirmed that melatonin can delay the progression of advanced prostate cancer resistance by blocking the interaction between AR-V7 and NF-κB (Liu et al., 2017). This suggests that the combination of melatonin and hormone therapy may be a viable treatment option.

In addition to melatonin, the core biological clock gene is associated with the AR signaling pathway. One study revealed rhythmic changes in the expression of the core clock genes *Per1, Cry1, Bmal1* and *Rev-erbα* in normal prostate tissue (Cao et al., 2009). However, the expression of Per1 in prostate cancer is significantly reduced, and overexpression of Per1 can inhibit cancer cell growth. Furthermore, the expression of Per1 is positively regulated by androgens. After stimulation by dihydrotestosterone (DHT), Per1 expression is increased, and the forced upregulation of Per1 weakens expression of androgen-sensitive genes. This indicates that Per1 interacts with the AR signaling pathway and modulates AR-regulated genetic expression (Cao et al., 2009) (as shown in Figure 2). These findings reveal an important role of Per1 in the development of prostate cancer and provide new clues for studying mechanisms underlying the occurrence and treatment of prostate cancer.

Another study revealed that *CRY1*, a tumor-specific AR signaling pathway target gene, is involved in androgen-regulated DNA repair and plays an important role in the growth of androgen-resistant prostate cancer (CRPC). By regulating the timing of expression of DNA repair factors (ATM and CHK2), CRY1 promotes DNA repair and CRPC growth (Shafi et al., 2021) (as shown in Figure 2). This study reveals a new mechanism of action for CRY1 in response to genotoxic damage and offers potential therapeutic targets to improve patient outcomes.

RAR-related orphan receptor (ROR), a positive regulator of the clock rhythm regulation loop, has three subtypes, ROR-α, ROR-β and ROR-γ, and different expression patterns. ROR-α and ROR-β expression do not noticeably change in prostate cancer, while ROR-γ expression is closely related to tumor development and is the key determinant of *AR* gene expression. ROR-γ directly binds to the promoter RORE binding site and promotes *AR* transcription through the action of the nuclear co-activating factors SRC1 and SRC3 (as shown in Figure 2). Moreover, a small-molecule antagonist of ROR-γ can disrupt the interaction between ROR-γ, SRC and AR to inhibit expression of the AR (Wang et al., 2016). The above results confirm that ROR-γ can act as a therapeutic target alone or in combination with other factors in the development of prostate cancer.

The Simon Linder team at the Netherlands Research Institute carried out a comprehensive polynomial analysis of tissues isolated after 3 months of AR-targeted enzalutamide monotherapy in patients with high-risk prostate cancer (Linder et al., 2022). Transcription analysis revealed that AR inhibition led to the development of neurosecretory-like conditions in tumors. In addition, epigenome analysis revealed reprogramming of a large number of genes encoding the enzalutamide-induced pioneer factor FOXA1 from an inactive chromosome site to an active synchronous regulating element that controls survival signals. Notably, treatment-induced FOXA1 sites were enriched in ARNTL, the circadian clock component. ARNTL levels after treatment correlated with patient clinical outcomes. Knockout of *ARNTL* significantly reduced the growth of prostate cancer cells. This study revealed that ARNTL, a circadian regulator, was an acquired fragile site after AR inhibition (Linder et al., 2022), providing new clues for the treatment of prostate cancer resistance.

## 6 Chronotherapy and prostate cancer

Based on an in-depth understanding of the relationship between the circadian rhythm and the development of cancer, there is growing interest in the use of Chronotherapy to improve cancer treatment. A large number of studies have confirmed that more than 30 chemotherapy drugs can result in a greater than 50% reduction in efficacy due to differences in the duration of administration. In ovarian cancer, patients treated with doxorubicin in the evening and cisplatin in the morning showed more severe complications, requiring lower drug dosages and delayed treatment, when compared to those treated with doxorubicin in the morning and cisplatin in the evening (Hrushesky, 1985; Lévi et al., 1990). The effectiveness of paclitaxel, a first-line therapeutic drug for treating tumors, including prostate cancer, has also been shown to be rhythmic (Tang et al., 2017). Decreased expression of PER3 in prostate cancer stem cells can increase cancer cell resistance to paclitaxel (Cai et al., 2018). Restoring BMAL1 expression in tongue squamous cell carcinoma or administering paclitaxel at the peak of BMAL1 expression can improve paclitaxel sensitivity and better inhibit tumor proliferation (Tang et al., 2017). Trastuzumab is the only approved treatment for HER2-positive gastric cancer (von Arx et al., 2023), and its resistance is linked to hexokinase 2 (HK2)-controlled high-glucose enzymatic activity. HK2 has a day and night rhythm mode (ZT6(Zeitgeber time) has the highest expression, ZT18 has the lowest expression) and is regulated by a transcription factor composed of PPARγ and the clock gene, PER1. Studies have confirmed that combination of metformin and trastuzumab at ZT6 with an inhibitor of PER1 can significantly enhance the therapeutic effect of trastuzumab (Wang et al., 2022). These findings suggest the importance of introducing clock rhythms into tumor therapy and indicate that a potential time-course treatment strategy may reverse chemotherapy resistance.

Studies have shown that the circadian rhythm of prostate cancer patients who have not undergone androgen deprivation therapy (ADT) may be disrupted, and ADT treatment may restore function (Bartsch et al., 2009; Hanisch et al., 2011). A hot flash is the most common adverse reaction after prostate ADT treatment. A study by Laura J Hanisch *et al.* involving 47 patients receiving ADT for prostate cancer, collected data from two 24-h rhythmic cycles to investigate the relationship between ADT therapy and hot flashes (Hanisch and Gehrman, 2011). The results showed that after receiving ADT, the frequency of hot flashes increased, and a marked circadian rhythm was observed, with peak periods occurring earlier in the afternoon. In patients not treated with ADT, there were rhythmic disruptions, so the authors suggested that the increase in hot flashes may represent the normalization of circadian rhythms after ADT treatment (Hanisch and Gehrman, 2011). Proton beam therapy (PBT), as an effective treatment for localized prostate cancer, can cause adverse events such as worsening of lower urinary tract symptoms (LUTS). Researchers compared the differences in LUTS among patients receiving PBT treatment at different times of the day, and the results showed that morning PBT treatment significantly improved LUTS and quality of life compared to noon and evening treatments (Negoro et al., 2020). Another study assessed the impact of high-dose radiation therapy on disease control and related toxicity in patients with prostate cancer in a rhythm-changing manner during the day and night. The results showed that night-time radiation caused more severe intestinal complications than day-to-day radiation did, and the effects were worse in older patients, resulting in poor 5-year biochemical failure-free survival (Hsu et al., 2016). The above studies indicate that ADT treatment can restore the circadian rhythm of patients. On this basis, chemotherapy and radiation therapy at appropriate times may enhance treatment effects and improve disease management. Although there is currently a lack of research on circadian rhythm changes in prostate cancer and timing of drug treatment strategies, treatment based on the rhythm changes of drug efficacy may be an ideal form of patient tailored therapy.

## 7 Conclusion

As one of the major diseases affecting men, the exploration of the pathogenesis of prostate cancer remains underexplored. Although some progress has been made in clinical practice, targeted androgen therapy still cannot block the development of tumors. Epidemiological studies have shown that disrupted circadian rhythms lead to the development of prostate cancer, but the physiological mechanism linking circadian rhythms and prostate cancer is not clear. Preclinical studies have shown that changes in circadian rhythm regulatory factors are closely related to the growth of prostate cancer and can also affect the stemness and tumorigenicity of prostate cancer stem cells. Androgens and androgen receptor signaling pathways are involved in regulating various biological processes, such as the cell cycle, DNA damage repair, and cell metabolism, which are closely related to circadian rhythms. Research has confirmed that circadian rhythm genes are associated with the AR pathway, but the relationship between circadian rhythm regulatory circuits and AR signaling pathways has not been fully elucidated. Additionally, laboratory preclinical research evidence links changes in circadian rhythm regulatory factors with the growth of prostate cancer, promoting the development of prostate cancer by affecting processes such as glycolytic metabolism, the stemness and tumorigenicity of tumor stem cells, and DNA damage repair. However, the precise mechanisms by which the body clock influences these pathways remain to be determined. Some metabolic pathways also exhibit dynamic circadian rhythms, making the timing of treatment critical. This provides a theoretical basis for the application of chronotherapy in prostate cancer. Therefore, future research is expected to reveal the mechanisms of circadian rhythm regulatory circuits in prostate cancer, providing new treatment methods for improving patient quality of life, enhancing treatment effectiveness, and reducing treatment side effects.
